# The Central African Republic – A fragile centre: *Emergency preparedness through national, regional and One Health approaches*

**DOI:** 10.4102/jphia.v17i1.1941

**Published:** 2026-10-06

**Authors:** Denis Ako-Arrey, Frankline Sevidzem Wirsiy, Bonaventure Bazirutwabo, Jean Méthode Moyen

**Affiliations:** 1 Global Actions for Public Health Services (GAPS), Yaoundé, Cameroon; 2 Africa Centres for Disease Control and Prevention, Yaoundé, Cameroon; 3 Independent Consultant, Geneva, Switzerland; 4 Ministry of Health and Population, Bangui, Central African Republic

**Keywords:** The Central African Republic, emergency and pandemic prevention, preparedness and response, One Health, regional cooperation, health security, policy analysis

## Abstract

The Central African Republic (CAR) faces persistent public health emergencies driven by constrained health systems capacity, recurrent outbreaks and its geographic position at the intersection of six high-risk countries. Cross-border mobility, ecological interconnectedness and limited multisectoral coordination amplify vulnerability to epidemics and pandemics. Strengthening emergency preparedness in CAR has implications beyond national borders, given its central role within a highly connected regional epidemiological landscape. A structured qualitative policy analysis was conducted using peer-reviewed and grey literature (2015–2025). The analysis followed a three-stage framework: problem definition, identification of policy options and comparative evaluation. Three policy pathways were assessed using five criteria: technical feasibility, political and institutional feasibility, financial sustainability, responsiveness to cross-border risks and alignment with One Health principles. Three policy pathways were identified: national system strengthening, regional integration and a hybrid One Health model. National approaches improve domestic capacity but are insufficient for transboundary risks. Regional approaches enhance coordination but depend on sustained institutional alignment. The hybrid model demonstrates the strongest overall performance by integrating national capacity, regional cooperation and multisectoral coordination. Emergency preparedness in CAR requires a shift from nationally bounded strategies to integrated regional and multisectoral systems. A hybrid One Health model provides the most feasible and sustainable pathway to strengthen outbreak detection, preparedness and response.

## Introduction

The Central African Republic (CAR) is often described as the ‘beating heart’ of Africa, reflecting its geographic centrality rather than political or economic influence. This central position, however, represents less a strategic advantage than a structural vulnerability within a region characterised by conflict, population mobility, ecological interconnectedness and shared zoonotic risks, alongside recurrent public health threats.^1,2^ The Central African Republic borders Cameroon, Chad, Sudan, South Sudan, the Democratic Republic of the Congo and the Republic of Congo, placing it at the centre of a highly integrated regional epidemiological landscape. The Central African Republic continues to experience recurrent outbreaks of measles, cholera, meningitis and emerging zoonotic diseases.^3^ In 2020, more than 28 900 measles cases were reported nationally, with ongoing transmission in subsequent years.^4^ Cholera outbreaks linked to flooding events highlight the interaction between environmental shocks and public health vulnerability.^5^ Mortality patterns further highlight underlying system constraints, with communicable diseases and nutritional deficiencies accounting for a substantial proportion of deaths, reflecting persistent gaps in access to essential health services and preventive care.^6,7^ These challenges are compounded by population displacement and environmental stressors. The Central African Republic hosts over 500 000 internally displaced persons, while climate variability has intensified food insecurity, affecting more than 2.6 million people.^8,9^ At the same time, livestock movement, wildlife interaction and shared ecosystems create conditions for zoonotic spillover.^10^ Importantly, these risks are not confined within national borders. Porous borders, informal crossings and socio-economic interdependence facilitate the rapid spread of infectious diseases across the region.^11^ As such, emergency preparedness in CAR cannot be effectively addressed through nationally bounded approaches alone. This paper applies a structured policy analysis to examine alternative strategies for strengthening emergency preparedness in CAR. It evaluates national, regional and hybrid approaches through a One Health lens, assessing the extent to which each pathway addresses multisectoral coordination and cross-border health risks. The objective is to identify a context-appropriate policy pathway that aligns with CAR’s epidemiological, institutional and regional realities.

## Research methods and design

### Study design and analytical framework

This study employed a structured qualitative policy analysis to examine emergency preparedness strategies in CAR. The analysis was designed to systematically evaluate policy pathways in relation to CAR’s complex health security environment. A three-stage analytical framework adapted from established public policy and health policy analysis approaches was applied^12,13^:

- Problem definition – synthesis of evidence on health system capacity, epidemiological burden, surveillance and laboratory systems, population mobility and environmental risk drivers.
- Identification of policy options – derivation of plausible policy pathways based on national, regional and global health security frameworks.
- Comparative evaluation – structured assessment of policy options using predefined criteria.

This framework enabled a transparent and reproducible comparison of policy alternatives.

### Evidence identification and selection

A targeted evidence synthesis was conducted using peer-reviewed and grey literature published between 2015 and 2025 and available in English or French. Searches were performed across major academic databases (including PubMed, Scopus and Google Scholar) using combinations of keywords related to emergency preparedness, outbreak response, health security, surveillance systems, One Health, and cross-border health dynamics, with geographic relevance to CAR and the Central African sub-region. To ensure contextual relevance and reduce publication bias, grey literature was systematically included, drawing on institutional reports, policy documents and technical guidelines from the World Health Organization (WHO), Africa Centres for Disease Control and Prevention (Africa CDC), World Bank, The Food and Agriculture Organization of the United Nations (FAO), United Nations Children’s Fund (UNICEF), United Nations (UN) World Food Programme (WFP), United Nations High Commissioner for Refugees (UNHCR), and regional institutions such as Economic Community of Central African States (ECCAS).

### Data extraction and synthesis

Relevant data were extracted using a structured template capturing key domains, including health system capacity, surveillance performance, epidemiological trends, cross-border dynamics, environmental risks and policy implementation experiences. Given the heterogeneity of sources, a quantitative meta-analysis was not appropriate. Instead, a thematic synthesis approach was used to identify patterns, gaps and policy-relevant insights that informed both the problem definition and policy option development.

### Identification of policy options

Policy options were identified inductively from the literature and existing health security strategies. Three policy pathways were selected based on their relevance and representation of distinct strategic approaches:

- National system strengthening.
- Regional integration.
- Hybrid One Health model.

These options represent plausible and policy-relevant pathways for strengthening emergency preparedness in CAR.

### Analytical criteria for comparative evaluation

The policy options were evaluated using five criteria adapted from established health policy analysis and public policy evaluation frameworks^12,13,14^:

- Technical feasibility: Capacity for implementation given infrastructure, workforce and system constraints.
- Political and institutional feasibility: Alignment with governance structures and regional cooperation mechanisms.
- Financial sustainability: Long-term viability based on domestic and external financing.
- Responsiveness to cross-border risks: Ability to address mobility, porous borders and transboundary disease dynamics.
- Alignment with One Health principles: Degree of integration across human, animal and environmental health systems.

These five criteria served as the primary analytical framework for the comparative evaluation. To support a more detailed and policy-relevant assessment, each criterion was further operationalised into specific dimensions (e.g. implementation complexity, multisectoral coordination and expected outcomes), as reflected in the comparative analysis (Table 1).

**TABLE 1:** Comparative assessment of emergency preparedness policy options for the Central African Republic.

Evaluation criterion	Option 1: National system strengthening	Option 2: Regional integration model	Option 3: Hybrid One Health model
Core policy logic	Build internal preparedness capacity through national investments in surveillance, laboratories, workforce and service delivery	Treat preparedness as a regional public good requiring shared systems and joint response mechanisms	Combine national strengthening with regional cooperation and multisectoral One Health governance
Primary actors	Ministry of Health and Population, national public health authorities, district teams, development partners	CAR government, neighbouring countries, Africa CDC, ECCAS, OCEAC, WHO AFRO and regional laboratories	Health, livestock and environment ministries; regional institutions; communities; humanitarian actors
Technical feasibility	Moderate (constrained by workforce, infrastructure and insecurity)	Moderate–high (existing platforms, but requires harmonisation)	High if phased (builds on national systems while linking to regional networks)
Political and institutional feasibility	High (aligned with national planning and sovereignty)	Moderate (requires coordination, trust and agreements)	Moderate–high (maintains national ownership while enabling coordination)
Financial sustainability	Low–moderate (high reliance on external funding)	Moderate–high (cost-sharing and pooled resources)	High (combines domestic prioritisation, partner funding and regional mechanisms)
Responsiveness to cross-border threats	Limited (focus remains within national borders)	High (directly addresses cross-border transmission)	Very high (integrates border health, community surveillance and regional alerts)
One Health integration	Limited (fragmented, sector-specific)	Moderate (partial integration across sectors)	Strong (systematic integration across human, animal and environmental health systems)
Multisectoral coordination	Limited	Moderate	Strong
Use of existing CAR strengths	Moderate (can strengthen facilities and workforce)	Moderate (links national systems to regional networks)	High (formalises community networks, local actors and informal systems)
Implementation complexity	Moderate (primarily national planning)	High (requires legal frameworks, diplomacy and coordination)	Moderate–high (more complex but can be phased)
Expected short-term benefits	Improved facility readiness, reporting and workforce capacity	Faster regional alerts and coordinated cross-border response	Improved early warning in high-risk districts and stronger coordination
Expected long-term benefits	Stronger domestic system, but continued exposure to external risks	Improved regional preparedness, but dependent on political continuity	Integrated and sustainable preparedness architecture across levels and sectors
Main risk	Reinforces national silos; insufficient for transboundary threats	Dependent on sustained regional cooperation	Requires strong coordination to avoid fragmentation
Overall assessment	Necessary but insufficient	Important but insufficient alone	Preferred option due to the balance of feasibility, sustainability and impact

CAR, Central African Republic; CDC, Centres for Disease Control and Prevention; ECCAS, Economic Community of Central African States; WHO AFRO, World Health Organization Regional Office for Africa; OCEAC, Organization for the Coordination of the Fight Against Endemic Diseases in Central Africa.

### One Health as an analytical lens

A One Health framework was applied as a central analytical lens in this study. One Health is defined as a multisectoral approach integrating human, animal and environmental health systems to address complex public health risks.^15^ In this analysis, One Health was not treated as a standalone policy option but as a cross-cutting framework used to assess the degree of multisectoral integration within each policy pathway. This approach allowed for a systematic evaluation of how different policy options incorporate coordination across sectors and governance levels in the context of emergency preparedness.

## Problem definition: A high-risk and interconnected health security environment

### Regional and epidemiological risk landscape

The CAR operates within one of the most complex health security environments globally, where epidemic risks are shaped by both domestic fragility and regional dynamics.^11,16^ Its six neighbouring countries have experienced major recurrent outbreaks over the past decade, including Ebola virus disease in the Democratic Republic of the Congo, yellow fever in Cameroon, hepatitis E in Chad and recurrent meningitis across the Sahel.^17,18^ The region also remains a hotspot for zoonotic diseases such as mpox, anthrax and Rift Valley fever, reflecting ecological conditions that facilitate cross-border pathogen transmission.^10,19^ These risks are compounded by extensive cross-border mobility. Informal movement of populations – including pastoralists, traders and displaced communities – occurs across porous and often unregulated borders, enabling the rapid spread of infectious diseases.^8,11^ In this context, epidemic threats are not confined within national boundaries but are embedded within a shared regional risk landscape. The Central African Republic’s central geographic position, therefore, amplifies its exposure to transboundary health threats and increases its relevance to regional health security.^1,2^

### Health system constraints and preparedness gaps

The Central African Republic’s ability to respond to these risks is constrained by limitations in the health system. According to the 2023 WHO Health Resources Availability Monitoring System (HeRAMS), only 48% of 1086 health facilities were fully functional, indicating that more than half are unable to reliably deliver essential services or support outbreak response.^20^ Health workforce density remains among the lowest globally, at approximately 1.3 health workers per 10 000 population, with stark disparities between urban and rural areas.^21^ Beyond increasing the availability of health workers, preparedness also requires strengthening the competencies of the existing workforce through continuous training in surveillance, outbreak response and One Health principles to enable effective multisectoral emergency preparedness and response. Surveillance systems remain incomplete and unevenly implemented. Only 54% of indicator-based surveillance reports were submitted on time in 2022, and event-based and community-based surveillance systems are not fully institutionalised.^22^ Laboratory capacity is similarly constrained. The Central African Republic has only two central laboratories that meet basic international biosafety standards, and most peripheral facilities lack capacity for bacteriology, molecular diagnostics and reliable specimen transport.^18^ These limitations result in delays in outbreak detection and confirmation, often requiring reliance on external reference laboratories.^18^ The epidemiological burden reflects these structural weaknesses. The Central African Republic continues to experience recurrent outbreaks of epidemic-prone diseases, including measles, cholera, meningitis and maternal and neonatal tetanus.^3^ In 2020, 28 903 measles cases and 139 deaths were recorded nationwide, with continued transmission in subsequent years, particularly among children under five years.^4^ Cholera re-emerged in 2022 along riverine trade routes, highlighting persistent vulnerabilities in water and sanitation systems.^5,23^ Sporadic cases of zoonotic diseases, including mpox, continue to be reported in forested regions, where surveillance and genomic capacity are limited.^3,19^ These challenges are further compounded by financing constraints. Domestic spending on epidemic and pandemic preparedness remains low, accounting for less than 5% of total health expenditure, resulting in high dependence on external funding and limiting long-term sustainability.^24,25^

### Ecological, climatic and mobility drivers of risk

The Central African Republic’s vulnerability is reinforced by ecological and climatic conditions that facilitate disease transmission. The country’s forests, wetlands and savannahs form a continuous ecological belt with neighbouring countries, creating ideal conditions for vector-borne and zoonotic diseases.^10,26^ Livestock mobility across pastoral corridors further complicates surveillance, as movements frequently bypass formal points of entry, limiting both veterinary and public health monitoring.^19^ Human–animal–environment interactions are particularly pronounced in forested areas, where reliance on bushmeat and close contact with wildlife increase the risk of zoonotic spillover.^10^ These dynamics are intensified by sub-optimal regulatory systems and limited integration between human and animal health sectors.^11,27^ Climate variability further exacerbates vulnerability, particularly through flooding, food insecurity and environmental disruption associated with changing climatic patterns.^28^ Recurrent flooding in southern and central regions displaces populations, disrupts health services and contaminates water sources, leading to outbreaks of cholera and other waterborne diseases.^5,26^ In 2022, over 1200 suspected cholera cases were reported following flooding events.^5^ At the same time, prolonged dry seasons and erratic rainfall contribute to food insecurity, with over 2.6 million people affected in 2024.^9^ Malnutrition weakens immune systems, increasing susceptibility to infectious diseases and compounding public health risks.^9^ Population displacement and mobility amplify these dynamics. The Central African Republic hosts more than 500 000 internally displaced persons, alongside refugees, returnees and mobile populations living in remote and underserved areas.^8^ Accessibility remains a major constraint, with several districts near international borders remaining difficult to reach, particularly during rainy seasons and periods of insecurity.^20^ These conditions create surveillance blind spots and delay response efforts.

### Systemic vulnerability and regional implications

Taken together, these factors illustrate a health system under continuous strain, characterised by recurrent outbreaks, limited preparedness capacity and vulnerabilities driven by ecological, climatic and socio-political factors.^11,16^ These challenges are not isolated to CAR but have broader regional implications. Given its central geographic position and high levels of cross-border interaction, health threats originating in CAR – or entering through its borders – can rapidly spread across neighbouring countries.^1,2^ At the same time, CAR remains highly exposed to outbreaks emerging elsewhere in the region.^17^ In the absence of integrated cross-border surveillance and coordinated response mechanisms, outbreaks may go undetected for extended periods, increasing the risk of regional spread.^11^ These vulnerabilities are not solely technical but structural. Health systems remain largely reactive rather than preventive, characterised by fragmented governance, weak multisectoral coordination and continued dependence on external financing for essential preparedness capacities.^29,30^ At the same time, the interconnected nature of CAR’s risk environment highlights the limitations of nationally bounded approaches to emergency preparedness. This context underscores the need for policy approaches that address not only domestic capacity gaps but also the regional and multisectoral drivers of health risk. It is within this complex and interconnected environment that alternative policy pathways for strengthening emergency preparedness must be assessed.

## Policy options for strengthening emergency preparedness and response

Building on the challenges outlined in the section ‘Problem definition: A high-risk and interconnected health security environment’, three policy pathways are considered to strengthen emergency preparedness in CAR. These options reflect distinct strategic approaches to addressing health system constraints, regional transmission dynamics and multisectoral risk drivers. While all three incorporate elements of One Health to varying degrees, they differ in the extent to which multisectoral coordination and cross-border integration are operationalised.

### Option 1: National system strengthening

This option focuses on strengthening CAR’s internal public health capacity as the primary mechanism for emergency preparedness. It prioritises investments in surveillance systems, laboratory infrastructure, workforce development, emergency operations, resilient medical supply chains and health facility functionality, addressing the critical service delivery gaps identified in section ‘Health system constraints and preparedness gaps’. Under this model, the Ministry of Health and Population would lead efforts to expand district surveillance teams, strengthen routine immunisation, improve laboratory diagnostic capacity and enhance emergency response systems. Development partners would provide technical and financial support, but planning and implementation would remain centred within national structures. This approach directly addresses the constraints identified in section ‘Health system constraints and preparedness gaps’, particularly gaps in health system capacity, surveillance performance and laboratory infrastructure. Strengthening these core functions is essential for improving early detection and response to outbreaks within CAR. However, this option has important limitations. As highlighted in sections ‘Regional and epidemiological risk landscape’ and ‘Ecological, climatic, and mobility drivers of risk’, epidemic risks in CAR are not confined within national borders. Cross-border mobility, shared ecosystems and livestock corridors create continuous pathways for disease transmission. A national-only approach may therefore improve domestic capacity while remaining insufficient to detect or respond effectively to transboundary threats. In addition, given CAR’s limited fiscal space, sustaining large-scale national investments without regional burden-sharing may prove challenging.

From a One Health perspective, this model may incorporate elements such as zoonotic surveillance or veterinary services, but these are typically implemented in a fragmented manner, with limited coordination across sectors.

### Option 2: Regional integration model

This option prioritises regional cooperation as the central strategy for emergency preparedness. It emphasises cross-border surveillance systems, shared data platforms, regional laboratory networks, joint outbreak investigations, coordinated vaccination campaigns and harmonised response protocols. Under this model, CAR would strengthen collaboration with neighbouring countries and regional institutions, including Africa CDC, ECCAS, Organization for the Coordination of the Fight Against Endemic Diseases in Central Africa (OCEAC), and the WHO Regional Office for Africa (AFRO). Preparedness efforts would be organised around cross-border risk corridors rather than solely national administrative boundaries. This approach responds directly to the regional and transboundary dynamics described in sections ‘Regional and epidemiological risk landscape’ and ‘Systemic vulnerability and regional implications’. By enabling countries to share information, coordinate alerts and pool resources through regional surveillance frameworks and strategies, regional integration can improve the timeliness and effectiveness of outbreak detection and response.^22,31^ Shared laboratory systems may also reduce delays in diagnostic confirmation, while joint simulation exercises can strengthen preparedness across borders. However, implementation challenges are significant. Effective regional integration requires political commitment, trust, data-sharing agreements, harmonised legal frameworks and sustained financing. In a context marked by conflict and institutional fragility, these conditions may be difficult to maintain consistently. Furthermore, as noted in section ‘Health system constraints and preparedness gaps’, CAR’s limited domestic capacity may constrain its ability to fully participate in and benefit from regional mechanisms. In terms of One Health integration, regional approaches may include cross-border zoonotic surveillance and environmental monitoring, but these are often unevenly implemented and remain primarily health-sector driven.

### Option 3: Hybrid One Health model

The hybrid One Health model combines targeted national strengthening with regional integration and systematic multisectoral coordination. It recognises that CAR requires stronger domestic systems, but that these must be embedded within regional and cross-sectoral frameworks to effectively address the drivers of health risk identified in section ‘Problem definition: A high-risk and interconnected health security environment’. Under this model, CAR would prioritise high-risk border districts for surveillance and strengthening, establish cross-border alert and information-sharing systems, and develop a tiered laboratory network linking peripheral facilities to national and regional reference laboratories. A national One Health coordination platform would be operationalised to facilitate joint risk assessment and response across human, animal and environmental sectors. Formal agreements with neighbouring countries would support coordinated outbreak response, synchronised interventions and resource sharing. This approach addresses the full range of challenges outlined in section ‘Problem definition: A high-risk and interconnected health security environment’. It strengthens core health system functions (section ‘Health system constraints and preparedness gaps’), responds to transboundary risk dynamics (sections ‘Regional and epidemiological risk landscape’ and ‘Systemic vulnerability and regional implications’), and integrates ecological and mobility-related drivers (section ‘Ecological, climatic, and mobility drivers of risk’) within a unified framework. Unlike the other options, the hybrid model systematically operationalises One Health by embedding multisectoral coordination into governance structures, surveillance systems and response mechanisms. It also builds on existing community networks and informal systems, which play a critical role in early detection and response in remote and underserved areas.^32^ While this model requires coordination across sectors and institutions, and may involve greater implementation complexity, it offers a more balanced and context-appropriate approach. By combining national capacity strengthening with regional cooperation and multisectoral integration, it aligns more closely with CAR’s risk profile and structural realities.

## Comparative policy analysis

The five core evaluation criteria outlined in the ‘Research methods and design’ section were operationalised into a broader set of dimensions to enable a more granular comparison of policy options.

### Narrative comparative analysis

The comparative analysis highlights that while each policy option addresses specific dimensions of CAR’s preparedness challenges, none is sufficient in isolation to address the full spectrum of risks outlined in section ‘Problem definition: A high-risk and interconnected health security environment’. The national system-strengthening approach is essential to addressing core health system gaps, including surveillance, laboratory capacity and workforce limitations. These investments are foundational for improving early detection and response within CAR. However, as demonstrated in sections ‘Regional and epidemiological risk landscape’ and ‘Ecological, climatic, and mobility drivers of risk’, epidemic risks are driven by cross-border mobility, shared ecosystems and regional transmission dynamics. A national-only approach may therefore improve internal capacity while leaving critical external drivers insufficiently addressed. The regional integration model responds more directly to the transboundary nature of these risks. By promoting shared surveillance systems, coordinated outbreak response and pooled resources, it enhances the capacity to detect and respond to cross-border threats. However, its effectiveness depends on sustained political commitment, harmonised regulatory frameworks and functional regional institutions. As noted in the section ‘Health system constraints and preparedness gaps’, CAR’s limited domestic capacity may also limit its ability to engage fully with and benefit from regional mechanisms. The hybrid One Health model combines the strengths of both approaches while addressing their limitations. By integrating targeted national investments with regional cooperation and multisectoral coordination, it aligns more closely with CAR’s risk profile. Importantly, this model does not treat One Health as a separate policy pathway but as a framework that can be variably integrated across options. While elements of One Health are present in all three approaches, they remain fragmented in the national and regional models. In contrast, the hybrid model systematically operationalises One Health by embedding coordination across human, animal and environmental health systems within both national and regional preparedness structures. Across the evaluation criteria, the hybrid model demonstrates the strongest overall performance. It balances technical feasibility with political and financial considerations while offering a more comprehensive response to cross-border and multisectoral risk drivers. Although more complex to implement, its phased, targeted nature enables incremental adoption, making it a context-appropriate and sustainable pathway to strengthen emergency preparedness in CAR.

## Discussion

This analysis demonstrates that emergency preparedness in the CAR cannot be effectively addressed through a solely national lens. The country’s risk profile is shaped not only by internal health system constraints but also by cross-border mobility, ecological continuity, population displacement and environmental change. As a result, preparedness must be understood as both a national responsibility and a regional and multisectoral challenge. A key conceptual clarification emerging from this study is the distinction between One Health as a framework and policy pathways as implementation strategies. One Health provides the conceptual basis for integrating human, animal and environmental health systems, but it is not itself a standalone policy option. Instead, it must be operationalised through concrete institutional arrangements, surveillance systems and governance mechanisms. The comparative analysis shows that while elements of One Health are present across all policy options, these remain fragmented in national and regional approaches. In contrast, the hybrid model provides a structured mechanism to embed multisectoral coordination within both national and regional preparedness systems. The findings also highlight that national system strengthening and regional integration should not be viewed as competing strategies. Strengthening domestic capacity is essential for improving surveillance, laboratory systems, workforce development and district-level response. At the same time, the regional nature of epidemic risks – driven by porous borders, informal mobility and shared ecosystems – requires coordinated approaches that extend beyond national boundaries. The hybrid model’s strength lies in its ability to connect these levels, linking national systems to regional mechanisms while addressing multisectoral risk drivers. Operationalising this approach requires moving from high-level commitments to implementation-focused design. Existing policy frameworks frequently emphasise the need for stronger surveillance, improved laboratories and better coordination, but these goals remain insufficient without clearly defined institutional arrangements, financing pathways and accountability mechanisms. In the context of CAR, this includes formalising cross-border agreements, strengthening specimen referral systems, establishing joint outbreak investigation protocols and integrating community-based surveillance into national systems. Regional cooperation emerges as a critical but underutilised component of preparedness. Platforms such as Africa CDC and the ECCAS provide opportunities for shared surveillance, coordinated response and resource pooling through emerging regional coordination mechanisms.^33^ However, their effectiveness depends on sustained political commitment, harmonised regulatory frameworks and integration into national systems. Without these elements, regional mechanisms risk remaining aspirational rather than operational. Importantly, this analysis underscores the value of a strengths-based perspective. While CAR is often characterised primarily in terms of fragility, existing capacities – particularly at the community level – play a crucial role in early detection and response. Community networks, local leaders, pastoralist associations and humanitarian actors frequently serve as the first point of contact for emerging health threats, especially in remote and insecure areas. Formalising and integrating these actors into surveillance and risk communication systems can enhance both effectiveness and trust. The hybrid model is not without challenges. It requires coordination across multiple ministries, alignment between national and regional institutions, and sustained financing. However, its implementation can be phased and targeted. Initial efforts can focus on high-risk border districts, priority disease threats and strengthening core functions such as surveillance, specimen transport and multisectoral coordination. Over time, these efforts can be expanded to include broader regional integration, advanced laboratory capacity and routine cross-border preparedness activities. Overall, the findings suggest that the most effective pathway for CAR lies not in selecting a single strategy, but in integrating national, regional and multisectoral approaches into a coherent preparedness architecture. By aligning investments with the country’s risk profile and leveraging both domestic and regional capacities, the hybrid One Health model offers a practical and context-appropriate framework for strengthening emergency preparedness. This study has some limitations. Firstly, the analysis relies on secondary data, including peer-reviewed and grey literature, which may be subject to reporting bias and variability in data quality. Although primary research studies were included where available, no original data collection was conducted, limiting the ability to validate findings in real-time operational settings. Secondly, the heterogeneity of sources and study designs constrained the ability to conduct a quantitative synthesis. However, the use of a structured analytical framework and triangulation of multiple data sources strengthens the robustness and policy relevance of the findings.

### Policy recommendations

The findings of this analysis indicate that strengthening emergency preparedness in CAR requires a phased and operational approach that integrates national capacity building, regional coordination and multisectoral governance. The following recommendations translate the preferred hybrid One Health model into implementable actions.

#### Recommendation 1: Establish cross-border surveillance units in high-risk districts

The Central African Republic should prioritise the establishment of integrated surveillance units in high-risk border districts, particularly along corridors with significant population mobility and outbreak history. These units should combine indicator-based, event-based, community-based and animal health surveillance, linking district-level detection to national and regional reporting systems. Operationalisation should include identifying priority districts, training multidisciplinary surveillance teams, developing standard operating procedures for alert verification and reporting, and establishing communication channels with the corresponding districts in neighbouring countries. Community informants – including local leaders, pastoralist networks and humanitarian actors – should be formally integrated into early warning systems.

#### Recommendation 2: Develop a tiered national–regional laboratory network

The Central African Republic should establish a tiered laboratory system linking peripheral health facilities and district laboratories to national and regional reference laboratories. This network should prioritise timely outbreak confirmation, safe specimen transport and access to molecular diagnostics. Implementation should include mapping existing laboratory capacity, strengthening selected district laboratories for basic diagnostics, establishing a national specimen transport system and formalising referral agreements with regional laboratories for advanced testing and surge capacity. Integration of laboratory data into surveillance systems is essential to ensure a timely response.

#### Recommendation 3: Operationalise a national One Health coordination platform

A functional One Health platform should be established to coordinate preparedness and response across human, animal and environmental health sectors. This platform should move beyond periodic coordination meetings to a structured mechanism with a defined mandate, governance structure and accountability framework. Key functions should include joint risk assessment, integrated preparedness planning, multisectoral outbreak response coordination and routine simulation exercises. The platform should also support the development of national risk maps for zoonotic and climate-sensitive diseases and oversee monitoring of One Health indicators.

#### Recommendation 4: Formalise bilateral and regional preparedness agreements

The Central African Republic should strengthen cross-border preparedness through formal agreements with neighbouring countries. These agreements should define operational mechanisms for data sharing, outbreak notification, specimen referral, joint investigation and coordinated response in border areas. Priority actions include harmonising case definitions and reporting protocols, establishing procedures for cross-border specimen transport, conducting joint simulation exercises and developing mutual support arrangements for outbreak response. Regional institutions such as Africa CDC, ECCAS, and WHO AFRO can facilitate and support these processes.

#### Recommendation 5: Strengthen community-based surveillance as a core system component

Community-based surveillance should be formalised as a central pillar of preparedness, particularly in remote and insecure areas where formal systems are limited.^32^ Local actors – including community health workers, religious leaders, teachers, pastoralist representatives and refugee leaders – should be trained to identify and report priority health events. Implementation should include developing simple alert definitions, providing appropriate reporting tools, linking community alerts to district verification systems and ensuring feedback mechanisms to maintain trust and engagement. Integrating community networks into national surveillance systems will significantly enhance early detection capacity.

### Implementation considerations

These recommendations should be implemented in a phased manner, beginning with high-risk districts and priority diseases. Financing can be mobilised through a combination of domestic resources, regional initiatives and global financing mechanisms such as the Pandemic Fund, World Bank programmes and Global Fund health systems strengthening investments. Strong coordination across ministries, partners and regional institutions will be essential to ensure coherence and sustainability.

## Conclusion

The CAR occupies a central position within a region where health risks are shared across borders, ecosystems and populations. Its preparedness challenges cannot be understood solely as limitations of the national health system but must also be viewed in the context of regional mobility, ecological dynamics and multisectoral interactions. This policy analysis compared three strategic pathways: national system strengthening, regional integration and a hybrid One Health model. The findings indicate that while national and regional approaches are both necessary, neither is sufficient in isolation. National strengthening is essential for building core capacity, while regional integration is critical for addressing transboundary risks. The hybrid model provides the most effective pathway by connecting these approaches and embedding multisectoral coordination within a unified preparedness framework. Importantly, the analysis highlights that preparedness must shift from broad policy commitments to operational systems, consistent with emerging global preparedness assessment frameworks.^34^ Strengthening surveillance, laboratory capacity, workforce development and coordination mechanisms requires clearly defined institutional arrangements, sustainable financing and practical implementation strategies. In a context where pathogens, populations and environmental risks move across borders, preparedness must also extend beyond them. Strengthening CAR’s emergency preparedness is therefore not only a national priority but also a regional public health investment within a context of longstanding health system pressures and recurrent public health emergencies.^35^ Building resilient systems at the centre of the continent contributes to strengthening health security across Central Africa.

## References

[1] World Bank. Central African Republic [homepage on the Internet]. Washington, DC: World Bank; 2025 [cited 2026 Jul 17]. Available from: https://www.worldbank.org/en/country/centralafricanrepublic

[2] International Fund for Agricultural Development (IFAD). Central African Republic [homepage on the Internet]. Rome: IFAD; 2025 [cited 2026 Jul 17]. Available from: https://www.ifad.org/en/web/operations/w/country/central-african-republic

[3] World Health Organization (WHO). Disease outbreak news [homepage on the Internet]. Geneva: WHO; 2025 [cited 2026 Jul 17]. Available from: https://www.who.int/emergencies/disease-outbreak-news

[4] UNICEF, Central African Republic Humanitarian situation report No. 4, 31 December 2023 [homepage on the Internet]. New York: UNICEF; 2024 [cited 2026 Jul 17]. Available from: https://www.unicef.org/documents/central-african-republic-humanitarian-situation-report-no-4-31-december-2023

[5] United Nations Children’s Fund (UNICEF). Central African Republic situation reports [homepage on the Internet]. New York: UNICEF; 2022 [cited 2026 Jul 17]. Available from: https://www.unicef.org/appeals/car/situation-reports

[6] World Health Organization (WHO). Central African Republic: Health profile [homepage on the Internet]. Geneva: WHO; 2024 [cited 2026 Jul 17]. Available from: https://www.who.int/countries/caf

[7] Population Division, Department of Economic and Social Affairs, United Nations (UN). World population prospects 2022: Summary of results [homepage on the Internet]. New York: UN; 2022 [cited 2026 Jul 17]. Available from: https://www.un.org/development/desa/pd/content/World-Population-Prospects-2022

[8] United Nations High Commissioner for Refugees (UNHCR). Central African Republic [homepage on the Internet]. Geneva: UNHCR; 2024 [cited 2026 Jul 17]. Available from: https://www.unhcr.org/countries/central-african-republic

[9] World Food Programme (WFP). Central African Republic [homepage on the Internet]. Rome: WFP; 2024 [cited 2026 Jul 17]. Available from: https://www.wfp.org/countries/central-african-republic

[10] Food and Agriculture Organization of the United Nations (FAO). One Health [homepage on the Internet]. Rome: FAO; 2022 [cited 2026 Jul 17]. Available from: https://www.fao.org/one-health

[11] Africa Centres for Disease Control and Prevention (Africa CDC). Framework for One Health practice in national public health institutes [homepage on the Internet]. Addis Ababa: Africa CDC; 2020 [cited 2026 Jul 17]. Available from: https://africacdc.org/download/framework-for-one-health-practice-in-national-public-health-institutes/

[12] Bardach E, Patashnik E. A practical guide for policy analysis: The eightfold path to more effective problem solving. 6th ed. Thousand Oaks, CA: CQ Press; 2020.

[13] Walt G, Gilson L. Reforming the health sector in developing countries: The central role of policy analysis. Health Policy Plan. 1994;9(4):353–370. 10.1093/heapol/9.4.353 10139469

[14] World Health Organization (WHO). Health policy and systems research: A methodology reader [homepage on the Internet]. Geneva: WHO; 2012 [cited 2026 Jul 17]. Available from: https://iris.who.int/

[15] Centers for Disease Control and Prevention (CDC). One Health [homepage on the Internet]. Atlanta, GA: CDC; 2022 [cited 2026 Jul 17]. Available from: https://www.cdc.gov/one-health/

[16] World Health Organization (WHO). Central African Republic: Country profile [homepage on the Internet]. Geneva: WHO; 2023 [cited 2026 Jul 17]. Available from: https://www.who.int/countries/caf

[17] WHO Regional Office for Africa (WHO/AFRO). Weekly bulletins on outbreaks and other emergencies [homepage on the Internet]. Brazzaville: WHO/AFRO; 2023 [cited 2026 Jul 17]. Available from: https://www.afro.who.int/health-topics/disease-outbreaks/outbreaks-and-other-emergencies-updates?page=11

[18] World Health Organization (WHO). Évaluation externe conjointe des principales capacités du Règlement sanitaire international (2005) de la République centrafricaine: Rapport de mission, 11–15 septembre 2023 [Joint external evaluation of the core capacities of the international health regulations (2005) of the Central African Republic: Mission report, 11–15 September 2023] [homepage on the Internet]. Geneva: WHO; 2024 [cited 2026 Jul 17]. Available from: https://www.who.int/fr/publications/i/item/9789240099340

[19] World Health Organization (WHO), Food and Agriculture Organization of the United Nations (FAO), United Nations Environment Programme (UNEP), World Organisation for Animal Health (WOAH). One Health joint plan of action (2022–2026): Working together for the health of humans, animals, plants and the environment [homepage on the Internet]. Geneva: WHO; 2022 [cited 2026 Jul 17]. Available from: https://www.who.int/publications/i/item/9789240059139

[20] World Health Organization (WHO). HeRAMS Central African Republic: Status update summary report September 2023 [homepage on the Internet]. Geneva: WHO; 2023 [cited 2026 Jul 17]. Available from: https://www.who.int/publications/m/item/herams-central-african-republic-status-update-summary-report-2023-09

[21] World Health Organization (WHO). Global health observatory: Global health workforce statistics [homepage on the Internet]. Geneva: WHO; 2023 [cited 2026 Jul 17]. Available from: https://www.who.int/data/gho/data/themes/topics/health-workforce

[22] WHO Regional Office for Africa (WHO/AFRO). Regional strategy for integrated disease surveillance and response – 2020–2030: Report of the Secretariat [homepage on the Internet]. Brazzaville: WHO/AFRO; 2019 [cited 2026 Jul 17]. Available from: https://iris.who.int/items/b28212c1-13a6-4097-9da2-2c45949c0fdf

[23] World Health Organization (WHO). Disease outbreak news: Cholera – Central African Republic [homepage on the Internet]. Geneva: WHO; 2022 [cited 2026 Jul 17]. Available from: https://www.who.int/emergencies/disease-outbreak-news

[24] The Pandemic Fund. The Pandemic Fund [homepage on the Internet]. Washington, DC: The Pandemic Fund; 2023 [cited 2026 Jul 17]. Available from: https://www.thepandemicfund.org/

[25] World Bank. From panic and neglect to investing in health security: Financing pandemic preparedness at a national level [homepage on the Internet]. Washington, DC: World Bank; 2017 [cited 2026 Jul 17]. Available from: https://openknowledge.worldbank.org/

[26] United Nations Environment Programme (UNEP). Emerging zoonotic diseases and links to ecosystem health – UNEP Frontiers 2016 chapter [homepage on the Internet]. Nairobi: UNEP; 2020 [cited 2026 Jul 17]. Available from: https://www.unep.org/resources/emerging-zoonotic-diseases-and-links-ecosystem-health-unep-frontiers-2016-chapter

[27] World Organisation for Animal Health (WOAH). Terrestrial animal health code [homepage on the Internet]. Current ed. Paris: WOAH. [cited 2026 Jul 17]. Available from: https://www.woah.org

[28] Intergovernmental Panel on Climate Change. Climate change 2022: Impacts, adaptation and vulnerability. Contribution of working group II to the sixth assessment report of the Intergovernmental Panel on Climate Change [homepage on the Internet]. Cambridge: Cambridge University Press; 2022 [cited 2026 Jul 17]. Available from: https://www.ipcc.ch/report/ar6/wg2/

[29] World Health Organization (WHO). Strengthening the global architecture for health emergency prevention, preparedness, response and resilience [homepage on the Internet]. Geneva: WHO; 2023 [cited 2026 Jul 17]. Available from: https://www.who.int/publications/m/item/strengthening-the-global-architecture-for-health-emergency-prevention--preparedness--response-and-resilience

[30] The Global Fund. Resilient and sustainable systems for health (RSSH) information note [homepage on the Internet]. Geneva: The Global Fund; 2023 [cited 2026 Jul 17]. Available from: https://www.theglobalfund.org

[31] Africa Centres for Disease Control and Prevention (Africa CDC). Africa CDC strategic plan 2023–2027 [homepage on the Internet]. Addis Ababa: Africa CDC; 2023 [cited 2026 Jul 17]. Available from: https://africacdc.org

[32] WHO Regional Office for Africa (WHO/AFRO). Technical Guidelines for IDSR, Third edition [homepage on the Internet]. Brazzaville: WHO/AFRO; 2019 [cited 2026 Jul 17]. Available from: https://www.afro.who.int/publications/technical-guidelines-integrated-disease-surveillance-and-response-african-region-third

[33] Africa Centres for Disease Control and Prevention (Africa CDC). The New Public Health Order: Africa’s health security agenda [homepage on the Internet]. Addis Ababa: Africa CDC; 2023 [cited 2026 Jul 17]. Available from: https://africacdc.org/news-item/the-new-public-health-order-africas-health-security-agenda/

[34] World Health Organization (WHO). Universal Health and Preparedness Review (UHPR) [homepage on the Internet]. Geneva: WHO; 2024 [cited 2026 Jul 17]. Available from: https://www.who.int/emergencies/operations/universal-health---preparedness-review

[35] Green A. The Central African Republic’s silent health crisis. Lancet. 2012;380(9846):964–965. 10.1016/S0140-6736(12)61523-2 22988578

